# Clinical impact of rapid influenza PCR in the adult emergency department on patient management, ED length of stay, and nosocomial infection rate

**DOI:** 10.1111/irv.12800

**Published:** 2020-08-26

**Authors:** David R. Peaper, Brittany Branson, Vivek Parwani, Andrew Ulrich, Marc J. Shapiro, Crystal Clemons, Melissa Campbell, Maureen Owen, Richard A. Martinello, Marie L. Landry

**Affiliations:** ^1^ Department of Laboratory Medicine Yale School of Medicine New Haven Connecticut USA; ^2^ Department of Radiology and Biomedical Imaging Yale School of Medicine New Haven Connecticut USA; ^3^ Clinical Redesign Yale New Haven Health New Haven Connecticut USA; ^4^ Department of Emergency Medicine Yale School of Medicine New Haven Connecticut USA; ^5^ Department of Pediatrics Division of Infectious Diseases Yale School of Medicine New Haven Connecticut USA; ^6^ Department of Infection Prevention Yale New Haven Health New Haven Connecticut USA; ^7^ Department of Laboratory Medicine Yale New Haven Hospital New Haven Connecticut USA; ^8^ Department of Internal Medicine Infectious Diseases Section Yale School of Medicine New Haven Connecticut USA; ^9^ Present address: Department of Emergency Medicine Harvard Medical School Boston Massachusetts USA

**Keywords:** bed management, ED length of stay, hospital‐acquired influenza, influenza PCR, nosocomial influenza, rapid PCR

## Abstract

**Background:**

Seasonal influenza causes significant morbidity and mortality and incurs large economic costs. Influenza like illness is a common presenting concern to Emergency Departments (ED), and optimizing the diagnosis of influenza in the ED has the potential to positively affect patient management and outcomes. Therapeutic guidelines have been established to identify which patients most likely will benefit from anti‐viral therapy.

**Objectives:**

We assessed the impact of rapid influenza PCR testing of ED patients on laboratory result generation and patient management across two influenza seasons.

**Methods:**

A pre‐post study was performed following a multifaceted clinical redesign including the implementation of rapid influenza PCR at three diverse EDs comparing the 2016‐2017 and 2017‐2018 influenza seasons. Testing parameters including turn‐around‐time and diagnostic efficiency were measured along with rates of bed transfers, hospital‐acquired (HA) influenza, and ED length of stay (LOS).

**Results:**

More testing of discharged patients was performed in the post‐intervention period, but influenza rates were the same. Identification of influenza‐positive patients was significantly faster, and there was faster and more appropriate prescription of anti‐influenza medication. There were no differences in bed transfer rates or HA influenza, but ED LOS was reduced by 74 minutes following clinical redesign.

**Conclusions:**

Multifaceted clinical redesign to optimize ED workflow incorporating rapid influenza PCR testing can be successfully deployed across different ED environments. Adoption of rapid influenza PCR can streamline testing and improve antiviral stewardship and ED workflow including reducing LOS. Further study is needed to determine if other outcomes including bed transfers and rates of HA influenza can be affected by improved testing practices.

## 1 INTRODUCTION

Influenza remains a global health care burden. The average estimated cost of seasonal influenza in the United States is $11.2 billion annually.^1^ Influenza infection can lead to severe morbidity and mortality among all patients, but the very young, the elderly, pregnant women, and persons with underlying disease are at higher risk. The efficacy of vaccination, our main defense against influenza, is variable and suboptimal. During influenza season, outpatient and emergency department visits increase as patients seek care. In symptomatic patients, a rapid and accurate influenza diagnosis can improve management.^2^, ^3^

Delayed influenza diagnosis in the ED can adversely impact patient management. Hospital admission may be delayed while awaiting test information for bed assignment. Appropriate infection prevention measures are needed to reduce hospital‐acquired (HA) influenza infections, a significant contributor to patient morbidity/mortality and healthcare costs.^4^, ^5^ Additionally, longer boarding times in the ED can lead to higher mortality and longer hospital length of stay (LOS).^6^ Furthermore, delays in diagnosis can delay initiation of antiviral therapy beyond the recommended 48 hours after symptom onset. Finally, for non‐admitted patients, lack of influenza test results before ED discharge can contribute to unnecessary antibiotic prescriptions.^7^

Several laboratory methods are available to diagnose influenza, but historically there has been a trade‐off between turn‐around‐time (TAT) and sensitivity. Less sensitive rapid influenza diagnostic tests (RIDTs) and direct fluorescent antigen testing (DFA) generate results within 15 minutes up to a few hours, while more sensitive nucleic acid amplified tests (NAAT) have substantially longer TAT, having typically been performed once a day in the local laboratory or sent to a reference laboratory. At our institution, methods for influenza diagnosis have historically varied depending on patient location and time of day.

While RIDTs can improve patient management in the ED,^3^ poor sensitivity, especially in adults, has propelled a transition to NAAT especially for hospitalized patients.^8^ Recently, several highly sensitive NAAT assays have become available that can be performed on demand with minimal hands‐on time and short in‐laboratory TAT. Some of these are available as point‐of‐care tests (POCT), and some detect pathogens in addition to influenza.

We sought to optimize influenza testing practices among three EDs and to provide a PCR result within 1 hour of sample receipt in the laboratory, regardless of time of day, to streamline patient admissions, promote timely and appropriate use of anti‐influenza medications, and hopefully reduce nosocomial transmission. We undertook a clinical redesign with stakeholders from emergency medicine, laboratory medicine, infection prevention, and bed management and implemented a series of changes during the 2017‐2018 influenza season. We identified key metrics including test volume, test TAT, ED LOS, oseltamivir prescriptions, bed transfers, and rate of HA influenza infection to assess program effectiveness.

## 2 METHODS

### 2.1 Study design and study site

A pre‐post study was performed following multiple interventions, comparing LOS, oseltamivir prescription, bed transfers, and rates of HA influenza infection between the pre‐intervention 2016‐2017 and post‐intervention 2017‐2018 influenza seasons. All encounters at one of three EDs with influenza testing ordered between January 20 and April 30 of the 2016‐2017 and 2017‐2018 flu seasons were eligible for inclusion. For HA influenza rates, data from the entire influenza seasons were assessed.

Yale‐New Haven Hospital (YNHH) is an urban 1541 bed academic tertiary care hospital located in New Haven, CT. YNHH has a mandatory influenza vaccination requirement for all directly employed staff and non‐employed medical staff. YNHH operates EDs at three locations: YNHH main campus (Site 1), a smaller urban hospital approximately 0.5 miles from the main campus (Site 2), and a free standing community ED 15 miles from the main campus (Site 3). These three sites differ in size, population, and cachement area.

### 2.2 Clinical redesign intervention

A multidisciplinary team involving stakeholders from the emergency department, laboratory medicine, infection prevention, bed management, and hospital Clinical Redesign Team was formed and met weekly to standardize influenza testing practices among EDs to optimize appropriate use of rapid influenza test results for bed management, infection prevention, and identification of patients for whom anti‐influenza medications would be beneficial.

The following interventions were developed as a result of the clinical redesign process: implementation of a rapid influenza A/B PCR assay performed 24/7 at on‐site laboratories for all ED locations, ED staff education, modification of the ED “Quick Pick List” to promote rapid PCR ordering, creation of a direct sample tubing pathway to the microbiology laboratory at Site 1, and modification of bed management processes (see below).

### 2.3 Bed management

YNHH has a combination of single‐ and multi‐occupancy rooms. Patients with the same respiratory viruses can be cohorted, but once a patient is identified as requiring respiratory virus isolation, either the patient or roommate(s) are transferred to an appropriate room. During the 2016‐2017 influenza season, bed assignments incorporated positive RIDT and DFA results, if available. After the intervention in 2017‐2018, bed assignments for patients admitted through the ED were not made until results of rapid influenza PCR were available whenever possible.

### 2.4 Influenza testing

For the 2016‐2017 influenza season, RIDT (Veritor; BD Biosciences), DFA (SimulFluor Respiratory Screen, MilliporeSigma), influenza laboratory developed test (LDT) PCR, either alone or as part of a respiratory virus PCR panel (RVP), were orderable in the electronic medical record (EMR). The influenza LDT PCR used was developed by the US Centers for Disease Control and Prevention (CDC). All testing was performed in clinical laboratories and not as a point‐of‐care test (POCT). At Site 1, samples were sent to the core laboratory before forwarding for virological testing. At Sites 2 and 3, the laboratories are contiguous to the EDs. RIDT testing was available to Site 2 and Site 3 EDs 24/7, and at Site 1 from the hours of 10 pm and 7 am. Respiratory virus DFA testing was performed at Site 1 from 7 am to 10 pm and included influenza A, influenza B, respiratory syncytial virus (RSV), adenovirus, and parainfluenza viruses 1‐3.^9^ RVP was performed two to three times a day at Site 1 and included influenza A, influenza B, RSV, adenovirus, parainfluenza viruses 1‐3, human metapneumovirus and human rhinovirus. All components of the RVP were LDTs.^10^ The stand‐alone influenza LDT PCR was run on the same schedule as the RVP.^11^ Choice of testing was at the discretion of the ordering provider. When DFA, RVP, or influenza LDT PCR were ordered at Sites 2 and 3, samples were couriered to Site 1 for testing.

Following the clinical redesign process and intervention, a rapid, on demand influenza by PCR assay (Xpert Xpress Flu, Cepheid) was made available at all sites by 1/16/2018. The option to directly send specimens to microbiology/virology via pneumatic tube system was added at Site 1. Results at all sites were entered into the EMR and verified manually. Rapid PCR replaced RIDT, DFA, and stand‐alone influenza PCR for ED ordering; RVP remained available primarily for admitted patients. All rapid influenza PCR testing was performed on‐site in the local laboratories 24/7 as a STAT test, and the RVP was performed two to three times per day at the core virology laboratory at Site 1.

### 2.5 Data collection

Data were extracted from the EMR (EPIC Systems) by the Joint Data Analytics Team at YNHH and Yale University. All ED encounters during which influenza testing was ordered in the ED were extracted along with associated clinical and demographic data: patient medical record number, date of birth, gender, unique visit identification number, ED location, ED visit start time, ED discharge time, admission status, admission date and time, influenza test ordered, test specimen number, test order date and time, test collection date and time, test verification date and time, test result, oseltamivir prescription date and time, first bed transfer date and time, originating transfer unit, and receiving transfer unit.

Separate data extractions were performed to identify all ED visits regardless of influenza testing or order location, with associated ED visit data and all influenza tests with associated laboratory data.

Cases of HA influenza are tracked as part of routine infection prevention.

### 2.6 Exclusion criteria

The following exclusion criteria were applied to encounters: patient age <18 years, patient leaving the ED against medical advice or without being seen by a provider, patient dying while in the ED, patients triaged to psychiatry or obstetrics services, patients lacking complete demographic information, laboratory studies lacking complete order information, encounters with irresolvable or incomplete admission or triage information, and encounters with atypical laboratory order patterns. Atypical laboratory ordering patterns (29 total encounters) were considered visits with two or more tests ordered and/or specimens collected for which the order, collection, and/or resulting date/time stamps did not allow for clear calculation of time intervals under investigation. Due to differences in practice and workflow, the pediatric emergency department was not included in the clinical redesign process, and pediatric patients presenting to off‐site EDs were excluded for consistency across all sites.

### 2.7 Hospital‐acquired infections

All encounters where patients with laboratory confirmed influenza diagnosed using specimens collected greater than 72 hours after admission underwent chart review.^12^ Patients with onset of signs and symptoms of acute respiratory tract infection within the first 72 hours of hospitalization were considered community‐acquired (CA) influenza infection.

The rate of HA influenza was calculated as:

$$\left\{\frac{\text{Patients}\;\text{with}\;\text{HA}\;\;\text{Influenza}}{\text{Hospitalized}\;\text{Patients}\;\text{with}\;\text{Laboratory}\;\text{Proven}\;\text{Influenza}}\right\}\times 1000$$

for the time period in question.

### 2.8 Data analysis

Data were organized in Microsoft Excel, and inclusion and exclusion criteria were applied. Statistical analyses were performed using SPSS v26.0 (IBM). Categorical variables were subjected to chi‐square analysis by cross‐tabs with calculation of adjusted standardized residuals in SPSS. Continuous variables were compared between relevant groups by Mann‐Whitney *U* test. Figures were prepared with GraphPad Prism v8.2 (GraphPad). A *P* < .05 was considered statistically significant.

## 3 RESULTS

A total of 5272 encounters met the case‐finding criteria, and, after applying the exclusion criteria, 5118 encounters underwent further analysis (Table 1). There were 1489 encounters from the 2016‐2017 influenza season and 3629 encounters from the 2017‐2018 influenza season. There were no significant differences in the gender, age, and proportion tested at each ED site.

**TABLE 1:** Demographics and influenza testing information

	2016‐2017	2017‐2018	Significance (*P*)
Total	1489 (100.0%)	3629 (100.0%)	
Gender (Female (%))	890 (59.8%)	2069 (57%)	.71
Age (mean (SD))	60.2 (21.1)	59.4 (20.5)	.16
Presenting ED			
Site 1	994 (66.8%)	2312 (63.7%)	.85
Site 2	334 (22.4%)	915 (25.2%)	
Site 3	161 (10.8%)	402 (11.1%)	
Disposition			
Non‐ICU*	738 (49.6%)	1513 (41.7%)	<.001
Not admitted*	422 (28.3%)	1328 (36.6%)	
Observation	173 (11.6%)	452 (12.5%)	
ICU/Step down	156 (10.5%)	336 (9.3%)	
First influenza test			
DFA/RIDT first*	783 (52.6%)	(0%)	<.001
LDT PCR/RVP first*	706 (47.4%)	286 (7.9%)	
Rapid PCR first*	(0%)	3343 (92.1%)	
Overall influenza result			
Influenza positive	359 (24.1%)	812 (22.4%)	.187
Influenza negative	1130 (75.9%)	2817 (77.6%)	

Note

Age was compared by *t* test, and categorical variables were compared by chi‐Squared. A *P*‐value < .05 was considered significant. “Overall Influenza Result” considers all testing performed on included patients within 24 h of presentation to the ED. Asterisks (*) indicate cells that significantly differed from expected values following calculation of adjusted, standardized residuals calculated in SPSS.

Abbreviations: DFA, Direct Fluorescent Assay; ED, Emergency department; ICU, Intensive care unit; LDT, Lab‐Developed Test; PCR, Polymerase Chain Reaction; RIDT, Rapid Influenza Diagnostic Test; RVP, Respiratory Virus PCR Panel.

The overall disposition of patients significantly differed between the flu seasons, with more testing being performed on non‐admitted patients in 2017‐2018 (*P* < .001). As expected, the first test performed for each encounter significantly differed between the influenza seasons with antigen methods and RVP/LDT PCR being replaced by rapid influenza PCR in 2017‐2018 (*P* < .001). Finally, the approach to testing became more straightforward in season 2. After implementation of rapid influenza PCR, significantly more encounters involved only a single specimen undergoing a single test (eg, rapid influenza PCR only) rather than multiple tests per specimen (eg, RIDT or DFA followed by RVP or LDT PCR) (data not shown; *P* < .001). However, the overall rate of flu positivity among all encounters was the same between 2016‐2017 and 2017‐2018 (*P* = .187) with 24.1% and 22.4% of patients testing positive for influenza, respectively (Table 1).

Looking at all specimens tested within 24 hours of ED presentation, we found that delayed identification of influenza was significantly reduced during the 2017‐2018 influenza season (Table 2; *P* < .001). In 2016‐2017, 12.3% of all influenza identifications were delayed, while this was only 0.6% of all influenza identifications for 2017‐2018 after implementation of rapid influenza PCR.

**TABLE 2:** Comparative efficiency of influenza diagnostic testing

	2016 ‐ 2017	2017 ‐ 2018
First test positive	315 (87.7%)	807 (99.4%)
Later test positive	44 (12.3%)	5 (0.6%)
Total influenza positive	349 (100%)	812 (100%)

Note

Influenza‐negative patients were not included in the analysis. Specimens collected within 24 h of presentation to the ED were considered when determining influenza status. *P* < .001.

**FIGURE 1:**
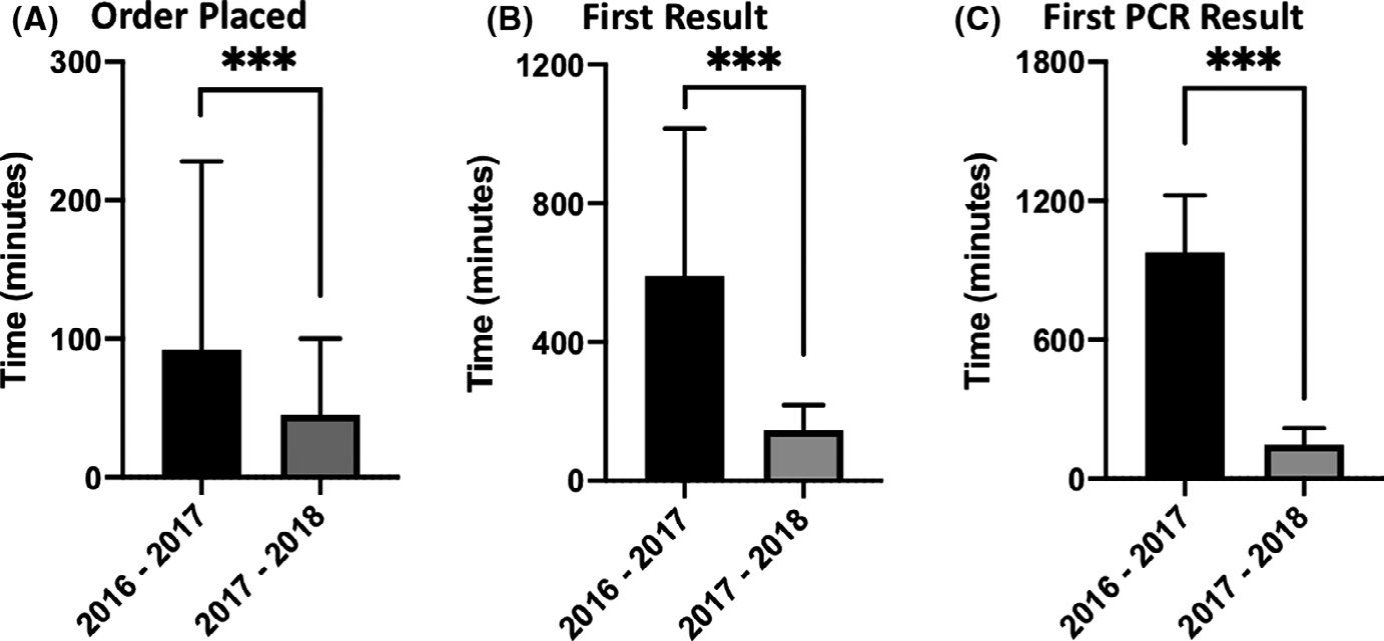
Time interval from arrival in ED to (A) order for influenza testing, (B) first result, and (C) first PCR result. Time of arrival in ED was considered *t* = 0 min. Median times in minutes and interquartile ranges are shown. Medians were compared by Mann‐Whitney *U* Test. *** indicates *P* < .001

Education was provided to ED providers during the 2017‐2018 influenza season to take advantage of rapid TAT to optimize care, and we compared order specific intervals between the seasons (Figure 1). The median time to order entry was significantly reduced from 92 minutes in 2016‐2017 to 45 minutes in 2017‐2018 (Figure 1A). There were more pronounced differences in the median time to first result and median time to first PCR result between the 2 years (Figure 1B,C). The median times to first result were 591 and 145 minutes, while median times to first PCR were 977 and 145 minutes for 2016‐2017 and 2017‐2018, respectively. All of these changes were statistically significant, and in 2017‐2018, 82.9% of visits had a PCR result available before the end of the ED visit compared to 36.7% in 2016‐2017 (*P* < .001, Data Not Shown).

**FIGURE 2:**
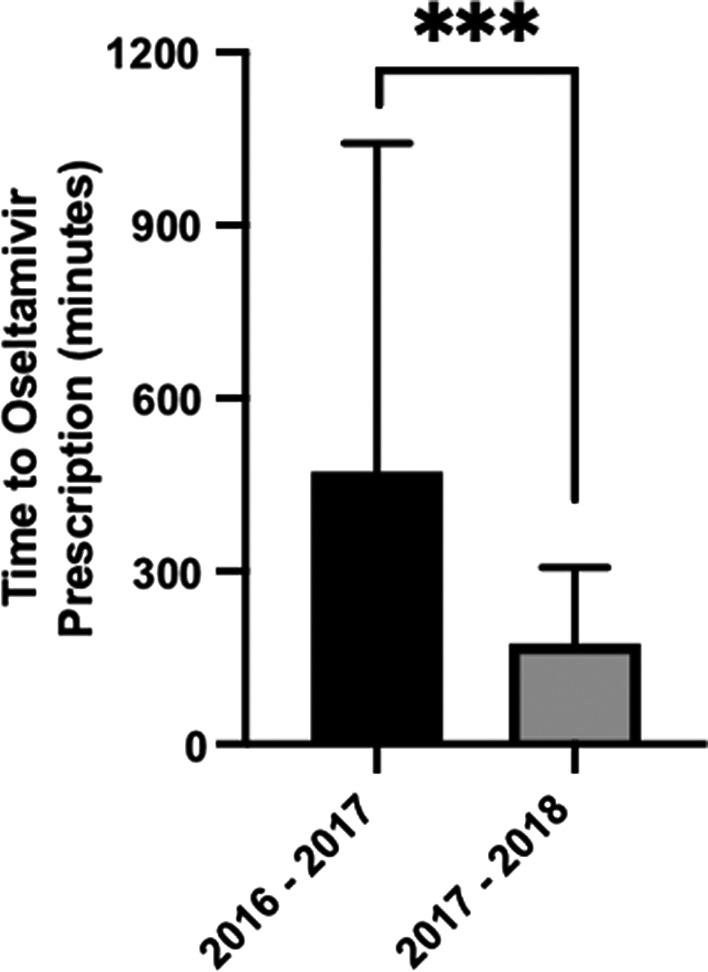
Time to oseltamivir prescription. The median time to oseltamivir prescription in minutes and interquartile range is shown. Time of arrival in ED was considered *t* = 0 min. Medians were significantly different when compared by Mann‐Whitney *U* Test (*** indicates *P* < .001)

We next examined rates of oseltamivir prescription among patients tested for influenza in the ED. There were significant treatment differences among the two influenza seasons for patients testing positive or negative for influenza (Table 3). Rates of empiric oseltamivir prescriptions in patients without influenza were higher during the 2016‐2017 influenza season with 3.9% of influenza‐negative patients having oseltamivir prescribed compared to 1.7% of influenza‐negative patients in 2017‐2018. Conversely, rates of oseltamivir prescription among patients with influenza were higher in 2017‐2018 with 71.4% of influenza‐positive patients having oseltamivir prescribed that year compared to 63.2% for 2016‐2017. Importantly, the time to oseltamivir prescription was significantly reduced following implementation of the rapid influenza PCR (Figure 2, *P* < .001). The median time to oseltamivir prescription was 174.6 minutes in 2017‐2018 compared to 472.2 minutes in 2016‐2017.

**TABLE 3:** Rates of oseltamivir prescription among patients tested for influenza

	2016‐2017	2017‐2018
Influenza negative		
No oseltamivir prescription	1086 (96.1%)	2769 (98.3%)
Yes oseltamivir prescription	44 (3.9%)	48 (1.7%)
Total influenza negative	1130 (100%)	2817 (100%)
Influenza positive		
No oseltamivir prescription	132 (36.8%)	232 (28.6%)
Yes oseltamivir prescription	227 (63.2%)	580 (71.4%)
Total influenza positive	359 (100%)	812 (100%)

Note

Data include all oseltamivir prescriptions entered in the electronic medical record for both admitted and non‐admitted patients. Rates of oseltamivir prescription were significantly different for both influenza‐negative (*P* < .001) and influenza‐positive (*P* < .001) patients between the two influenza seasons.

Our hospital has many multi‐bed rooms, and when accurate test results are not available before admission, influenza‐infected patients may be placed in rooms with uninfected patients, if other bed options are not available‐ increasing the risk for HA influenza transmission. A major source of patient dissatisfaction is bed transfers, and more rapid availability of influenza testing results could lead to fewer patient transfers. Unfortunately, among admitted patients, there was not a significant difference in transfers within 24 hours to and from rooms of the same acuity (Table 4). During the 2016‐2017 influenza season, 7.0% of patients had lateral transfers, and 7.3% of admitted patients had lateral transfers in the 2017‐2018 influenza season.

**TABLE 4:** Lateral transfers among admitted patients

	2016‐2017	2017‐2018
No transfer w/in 24 h	831 (93.0%)	1713 (92.6%)
Yes transfer w/in 24 h	63 (7.0%)	136 (7.4%)
Grand total	894 (100%)	1849 (100%)

Note

“Yes” includes only transfers of the same acuity. De‐escalation from an ICU unit to a non‐ICU unit was considered “No” transfer. Data does not include “Not Admitted” patients or patients placed in “Observation” (2016‐2017 n = 595, 2017‐2018 n = 1780). Data were not significant by Chi‐squared analysis (*P* = .814).

**FIGURE 3:**
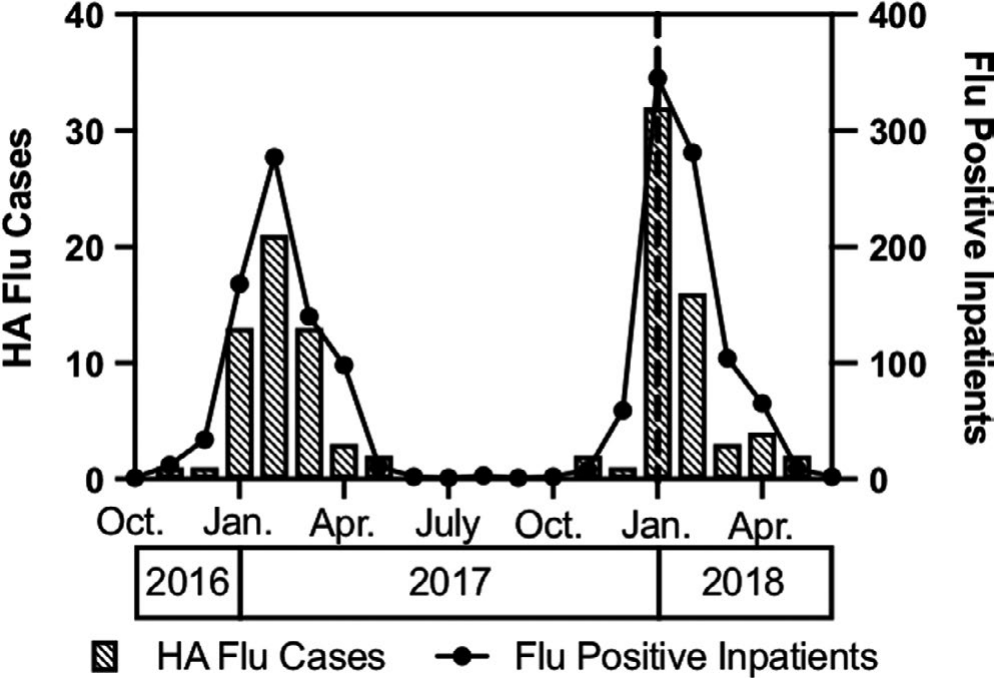
Hospital‐acquired (HA) and total inpatient influenza infections. HA influenza cases were defined as described in the Methods. Flu‐positive inpatients represent unique patients. The vertical dashed line (– –) indicates the month in which rapid influenza PCR was available at all three EDs

Rates of influenza positivity tracked regional and national trends (Figure 3), and months with the highest rates of influenza‐positive inpatients saw the highest rates of HA influenza. There were 52 and 58 patients found to have HA influenza with dates of diagnosis between October 1 and April 30 of the 2016‐2017 and 2017‐2018 influenza seasons, respectively. Over the same period, there were a total of 678 and 806 admitted patients with CA influenza, for a total of 730 and 864 patients with laboratory confirmed influenza, respectively. The overall rate of HA influenza for 2016‐2017 was 71.2 cases per 1000 influenza‐positive inpatients, and the rate for 2017‐2018 was 67.1. These rates did not significantly differ (*P* = .767). When cases identified after April 30 were included, there remained no significant differences (Data Not Shown). Additionally, HA influenza rates were not significantly different when calculated based upon the implementation of the rapid influenza PCR testing (Data Not Shown).

**FIGURE 4:**
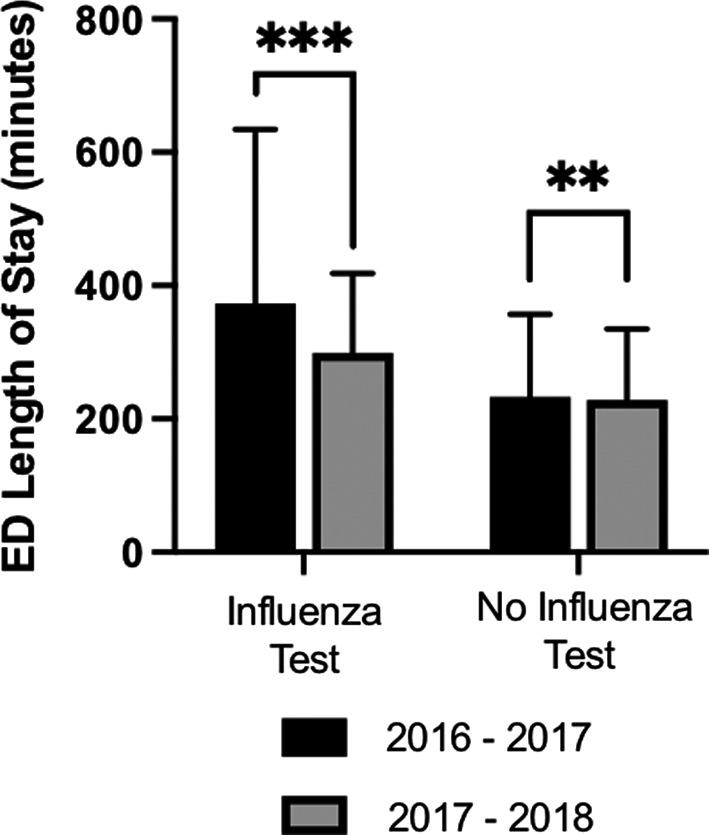
ED length of stay. Median lengths of stay in minutes and interquartile ranges are shown. Time of arrival in ED was considered *t* = 0 min. Medians were compared by Mann‐Whitney *U* Test. *** indicates *P* < .001, and ** indicates *P* < .01

We wanted to determine if changes in influenza testing practices and education could affect the LOS within the ED. We extracted relevant data from *all* ED visits during these time periods and compared the LOS of patients who underwent influenza testing and those who did not (Figure 4). A total of 90 719 ED visits met the inclusion criteria, with 44 459 and 46 170 visits in 2016‐2017 and 2017‐2018, respectively. Among these visits, 1489 and 3629 had testing for influenza ordered in the ED. The difference in median LOS between the two influenza seasons for patients *not* tested for influenza was 5 minutes (233 minutes vs 228 minutes for 2016‐2017 and 2017‐2018, respectively, *P* = .006). The difference in median LOS for visits with influenza testing between the two influenza seasons was 74 minutes (373 minutes vs 299 minutes for 2016‐2017 and 2017‐2018, respectively; *P* < .001).

## 4 DISCUSSION

We showed that implementation of rapid influenza PCR in conjunction with multidisciplinary clinical redesign and provider education can promote more expedient testing in the ED. This was successfully implemented across three EDs with different medical staff and laboratory capabilities. More importantly, we documented more timely and targeted administration of anti‐influenza medications and decreased ED LOS for patients undergoing influenza testing.

There was a significant decrease in the interval between ED triage and influenza test ordering in the 2017‐2018 cohort. This is likely a result of the education campaign raising awareness of influenza testing and the clinical redesign project. When combined with a rapid and sensitive PCR assay performed 24/7, the more rapid generation of results likely led to the positive outcomes we observed.

As expected and previously shown, patients with laboratory identified influenza were diagnosed more quickly following the implementation of rapid PCR testing.^2^, ^13^ However, previous studies often look at only in‐laboratory TAT,^2^ while we reported the times from patient arrival in the ED to order placement and result reporting. Institutions seeking to optimize testing practices and reduce global test TAT should consider interventions that promote prompt test ordering and specimen collection, submission, and testing.

There was no significant increase in influenza positivity rate following transition to rapid PCR. This was possibly due to the long‐standing availability of a sensitive LDT PCR at Site 1, albeit as a batched test. Previously, only RIDT was available at all three sites, so samples had to be couriered to Site 1 from Sites 2 and 3 for DFA or LDT PCR testing, adding to the longer TAT.

A greater percentage of influenza‐tested patients were discharged from the ED during the 2017‐2018 season (36.6% vs 28.3% in 2016‐2017), despite higher rates of hospitalization for influenza as a whole in the United States in the later season.^14^ While reasons for this difference were not explored, prior studies showed rapid NAAT testing in the ED was associated with a decrease in hospital admissions.^4^, ^15^ Additionally, some providers may not have previously ordered influenza testing, especially PCR testing, if results would not have been generated in a clinically actionable time‐frame for patients not requiring admission.

The EDs at our institution routinely operate above capacity, and any intervention promoting decreased LOS can affect ED throughput, patient satisfaction, and, potentially favorably, morbidity and mortality.^5^ Previous studies of influenza testing have not definitively shown a reduction in ED LOS.^15^, ^16^ In contrast, we found a significantly reduced ED LOS in influenza‐tested patients, with median ED LOS decreasing 74 mintues (19.8%). We also looked at an important control population over the same period to capture the effects of changes in ED workflow independent of influenza testing. We found a significant, but small, difference in the median LOS of patients not undergoing influenza testing over the same period, but for those patients, the median LOS decreased by only 5 minutes (2.1%).

Rapid testing can facilitate bed management decisions for admitted patients and improve infection prevention even in the ED among patients who will be discharged. However, we were unable to identify a difference in the rate of bed transfers between the two influenza seasons. There were a smaller number of transfers than anticipated. It is possible that many of the bed transfers in the hospital are for patients directly admitted rather than those passing through the ED. Alternatively, the contribution of respiratory viruses to bed transfers could have been overestimated. Additionally, our case finding would not capture the movement of influenza‐negative patients out of rooms with influenza‐positive patients.

We hypothesized that implementation of early diagnostic testing for influenza would facilitate rapid identification of infected patients expediting the implementation of appropriate infection control measures and bed management decisions to collectively decrease the rate of HA influenza. However, we did not find a significant difference in the rate of HA influenza between the two study periods.

Youngs et al^17^ reported that implementation of a comprehensive program including rapid influenza PCR testing reduced rates of HA flu. However, pre‐intervention rates of HA influenza in their study were higher than our rate at baseline, suggesting that there was greater opportunity for improvement. Additionally, at our institution, prior to the availability of rapid influenza PCR, many patients underwent testing for influenza by LDT PCR shortly after admission. Thus, infected patients may have been identified and placed on isolation relatively early in admission limiting patient‐to‐patient spread.

Identification of influenza‐positive patients earlier should decrease patient‐to‐patient transmission, but this only addresses one potential source for transmission. Changes to ED testing practices should not affect visitor‐to‐patient or staff‐to‐patient transmission of influenza. Notably, we had a policy requiring all staff to be vaccinated against influenza during the entirety of the 2016‐2018 study period. However, vaccinated individuals can shed influenza virus potentially making them a source of transmission.^18^ Additionally, medical and nursing staff may report to work with influenza like illnesses despite policies against such behavior.^19^

The 2017‐2018 North American influenza season was longer and more severe than the 2016‐2017 season. It is possible rapid PCR averted a higher HA influenza rate during 2017‐2018. It is also possible that we were unable to detect an effect on HA influenza due to an overall high burden of influenza. Additionally, awareness of 2017‐2018 influenza season severity may have changed clinicians’ influenza testing patterns. Lastly, this study was not prospectively powered to identify a difference between CA influenza and HA influenza cases. Future investigations including more seasons could provide insight into the impact of rapid testing on the prevention of HA influenza.

This study had several limitations. We looked at two different influenza seasons which varied in their duration and severity. The 2017‐2018 season had higher national levels of emergency department visits and hospitalization rates than 2016‐2017.^14^ This may partially explain the increased testing seen for non‐admitted patients in the 2017‐2018 cohort. Additionally, changes were not implemented at the beginning of the influenza season, and only partial influenza seasons were captured in our data. Implementation of these changes earlier in the 2017‐2018 season may have led to differences in HA influenza rates given the observed improvements in time to first result and time to oseltamivir prescription. However, rates of influenza testing among admitted patients were high during both influenza seasons, so these differences may have not been as pronounced.

While there is tremendous interest in the role of rapid diagnostic testing for antimicrobial stewardship, we did not assess the impact of rapid influenza testing on antibacterial usage. Additionally, we looked at rates of oseltamivir prescriptions rather than administration. However, this might be a more meaningful metric as it captures outpatients and inpatients through orders in the EMR.

Significantly more patients had influenza test results available prior to discharge from the ED after implementation of rapid PCR testing. Results were delayed in 17.1% of ED encounters, but 7.9% of encounters had LDT PCR instead of rapid PCR first. Other result delays were due to test ordering delays, incorrect test orders requiring manual correction, delayed specimen transport, instrument failures necessitating repeat testing, and inefficient result reporting due to the need for manual result entry. Improvements implemented in the 2018‐2019 flu season have further reduced ordering and transport errors. In addition, all rapid PCR instruments are now interfaced to the EMR, eliminating time delays due to manual entry, and autoverification has been implemented at Site 1. Workflow optimization, interfacing, and autoverification can significantly reduce in‐laboratory TAT for rapid PCR testing.^20^

In summary, clinical redesign featuring the implementation of a sensitive rapid influenza PCR led to a simplified testing algorithm, faster test TAT, more appropriate and earlier administration of antiviral therapy, and significantly shorter ED LOS. However, we found no impact on inpatient transfers and rates of HA influenza. No increase in influenza positivity was observed, likely due to prior availability of LDT PCR. Rapid, user‐friendly FDA‐cleared NAAT are significantly more expensive than RIDT, DFA, and LDT PCRs. To justify the added expense, improved patient outcomes should be documented. Our experience reinforced the critical importance multidisciplinary teams working together to reengineer workflows, streamline ordering, educate providers, and use rapid test results to promptly guide correct actions by caregivers. Even with the positive impacts observed in our study, opportunities for further improvements are evident and will be pursued in the future.

## CONFLICT OF INTEREST

Dr Martinello served on a scientific advisory board for Genetech for baloxivir. There are not other conflicts of interest.

## AUTHOR CONTRIBUTION

**David R. Peaper:** Conceptualization (equal); Data curation (lead); Formal analysis (lead); Visualization (lead); Writing‐original draft (equal); Writing‐review & editing (equal). **Brittany Branson:** Data curation (equal); Project administration (equal); Resources (equal); Writing‐original draft (equal); Writing‐review & editing (equal). **Vivek Parwani:** Conceptualization (supporting); Methodology (equal); Project administration (supporting); Resources (supporting). **Andrew Ulrich:** Conceptualization (supporting); Methodology (equal); Project administration (supporting); Resources (supporting). **Marc J. Shapiro:** Conceptualization (supporting); Methodology (equal); Project administration (supporting); Resources (supporting). **Crystal Clemons:** Project administration (lead); Resources (equal). **Melissa Campbell:** Data curation (equal); Investigation (equal). **Maureen Owen:** Project administration (supporting); Resources (equal); Validation (equal). **Richard Martinello:** Conceptualization (lead); Data curation (equal); Formal analysis (supporting); Investigation (equal); Methodology (equal); Supervision (equal); Writing‐review & editing (equal). **Marie Landry:** Conceptualization (lead); Investigation (equal); Methodology (equal); Supervision (lead); Validation (equal); Writing‐review & editing (equal).
